# Supplementary material for: Complete inclusion of bioactive molecules and particles in polydimethylsiloxane: a straightforward process under mild conditions
Source: Sci Rep. 2019 Nov 26;9:17575. doi: 10.1038/s41598-019-54155-5 (PMC6879495; doi:10.1038/s41598-019-54155-5)
Supplement: Supplementary file 1 — Supplemental Material [file 41598_2019_54155_MOESM1_ESM.docx]

**Supporting Information**

**Complete inclusion of bioactive molecules and particles in polydimethylsiloxane: a straightforward process for the under mild conditions**

Greta Faccio^a*^, Alice Cont^b,c^, Erik Mailand^a,d^, Elaheh Zare-Eelanjegh^a,e^, Riccardo Innocenti Malini^b^, Katharina Maniura-Weber^a^, René Michel Rossi^b^ and Fabrizio Spano^b,f*^

^a^ Empa, Swiss Federal Laboratories for Materials Science and Technology, Laboratory for Biointerfaces, Lerchenfeldstrasse 5, CH-9014 St. Gallen, Switzerland.

^b^ Empa, Swiss Federal Laboratories for Materials Science and Technology, Laboratory for Biomimetic Membranes and Textiles, Lerchenfeldstrasse 5, CH-9014 St. Gallen, Switzerland.

^c^ École Polytechnique Fédérale de Lausanne, Institute of Bioengineering and Global Health Institute, School of Life Sciences, CH-1015 Lausanne, Switzerland.

^d^ Ecole Polytechnique Fédérale de Lausanne, Institute of Mechanical Engineering and Bioengineering, CH-1015 Lausanne, Switzerland.

^e^ ETH Zurich, Institute for Biomedical Engineering, Laboratory of Biosensors and Bioelectronics (LBB), Gloriastrasse 35, ETZ F 75, CH-8092 Zurich, Switzerland.

^f^ ZHAW – Zurich University of Applied Sciences, Institute of Computational Physics, Technikumstrasse 9, CH-8401 Winterthur, Switzerland.

*E-mail: [fabrizio.spano@zhaw.ch](mailto:fabrizio.spano@zhaw.ch)

[greta.faccio@gmail.com](mailto:greta.faccio@gmail.com)


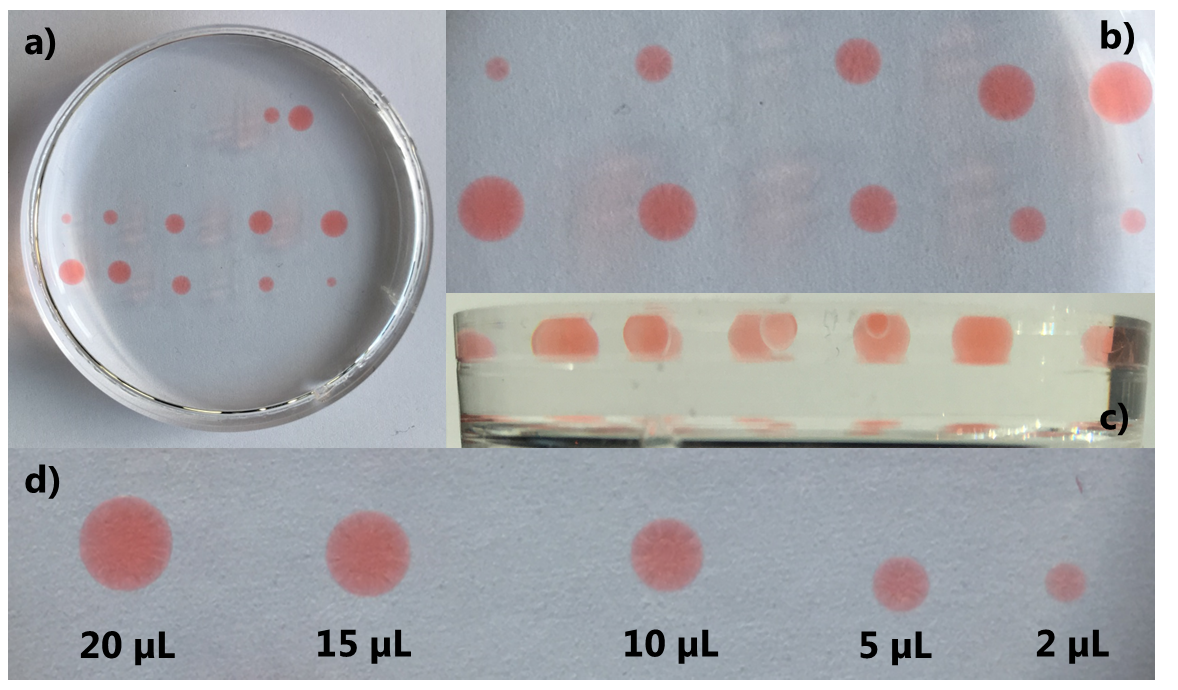


**Figure SI1.** Soft liquid composites sample made with inclusions of red dye loaded droplets: a) Top view of the sample; b) Zoomed view of the soft liquid composite sample illustrating the different droplet volumes; c) 90° side view of the sample illustrating the penetration of the red liquid droplets into the PDMS matrix. d) Zoomed top view illustrating the droplet volumes with 20, 15, 10, 5 and 2 µl, respectively.


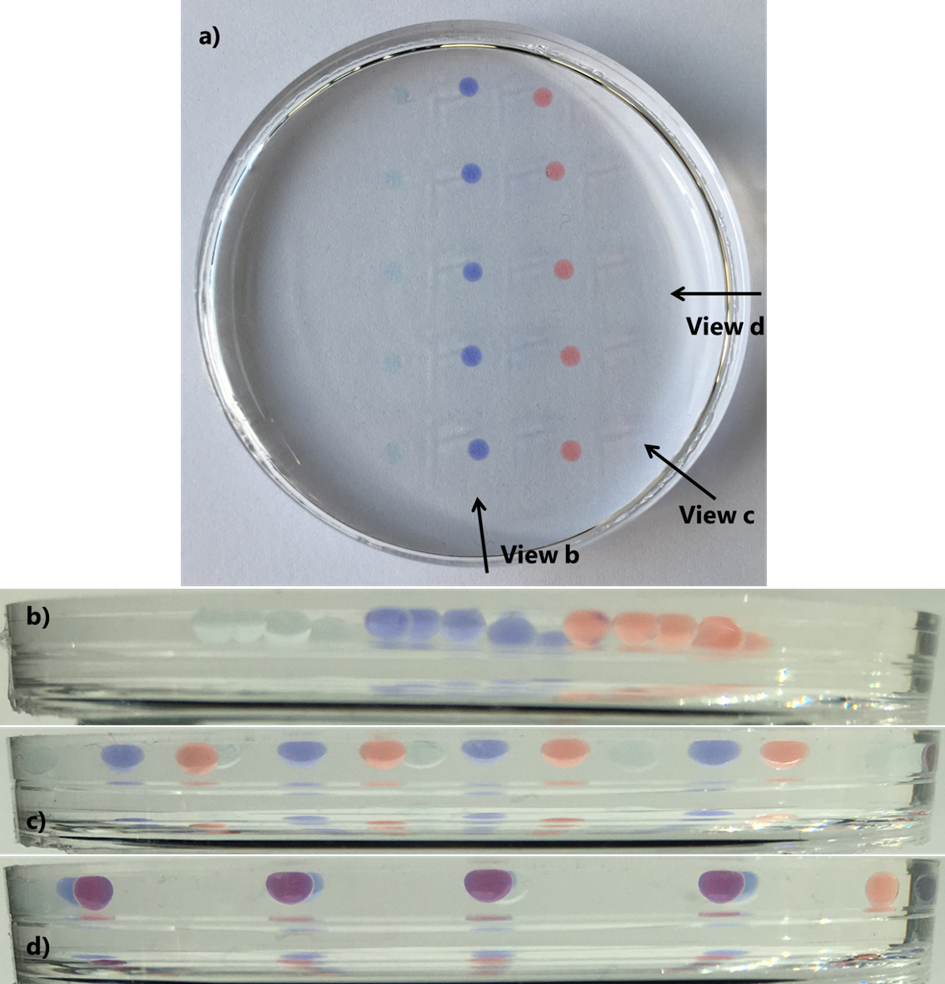


**Figure SI2.** Soft liquid composites sample made with inclusions using different dye-loaded droplets: a) Top view of the sample; b), c) and d) 90° side views of dye-loaded inclusions into the PDMS matrix changing colors due the superposition of the droplets in function of the selected lateral view angle.


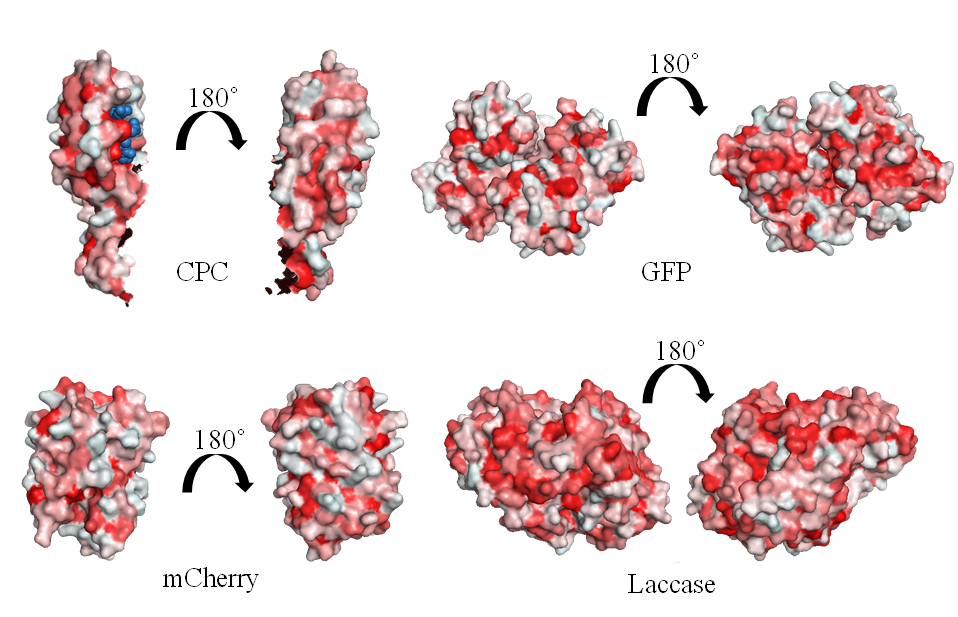


**Figure SI3.** Surface hydrophobicity of the proteins used in the study: CPC (with the bilin cofactor as blue spheres, PDB:4F0T), GFP (PDB:1EME), mCherry (PDB:2H5Q), and laccase (PDB:1KYA) visualized according to the normalized Eisenberg hydrophobicity scale showing hydrophilic regions as white and hydrophobic ones in red.


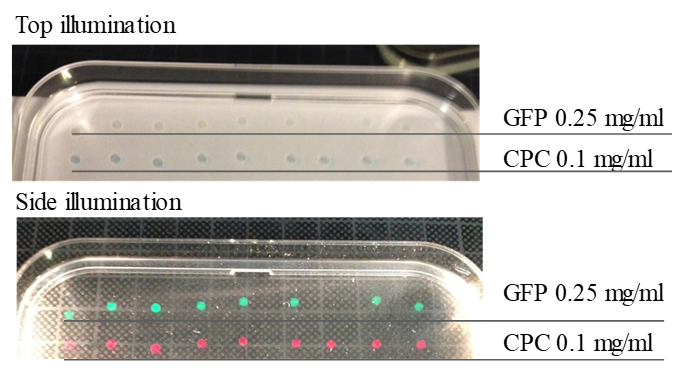


**Figure SI4.** Photographic imaging of fluorescent proteins GFP and CPC as liquid droplets in PDMS upon top and lateral illumination.
